# HSPB1 Gene Variants and Schizophrenia: A Case-Control Study in a Polish Population

**DOI:** 10.1155/2022/4933011

**Published:** 2022-03-15

**Authors:** Malgorzata Kowalczyk, Krzysztof Kucia, Aleksander Owczarek, Renata Suchanek-Raif, Monika Paul-Samojedny, Piotr Choreza, Jan Kowalski

**Affiliations:** ^1^Department of Medical Genetics, School of Pharmaceutical Sciences, Medical University of Silesia, Jednosci 8, 41-200 Sosnowiec, Poland; ^2^Department of Psychiatry and Psychotherapy, School of Medical Sciences, Medical University of Silesia, Katowice, Ziolowa 45, 40-635 Katowice, Poland; ^3^Health Promotion and Obesity Management Unit, Department of Pathophysiology, Faculty of Medical Sciences in Katowice, Medical University of Silesia, Katowice, Medykow 15, 40-752 Katowice, Poland; ^4^Division of Statistics, Department of Instrumental Analysis, School of Pharmaceutical Sciences, Medical University of Silesia, Ostrogorska 30, 41-200 Sosnowiec, Poland

## Abstract

Schizophrenia (SCZ) is a severe psychiatric disorder that has a significant genetic component. HSPB1 (HSP27) is known for its neuroprotective functions under stress conditions and appears to play an important role during the development of the central nervous system, which is in agreement with the neurodevelopmental hypothesis of SCZ. The aim of the present case-control study was to investigate whether *HSPB1* variants contribute to the risk and clinical features (age of onset, symptoms, and suicidal behavior) of SCZ in a Polish population. To the best of our knowledge, this is the first study that investigated the association between the *HSPB1* polymorphisms and SCZ. Three SNPs of *HSPB1* (rs2868370, rs2868371, and rs7459185) were genotyped in a total of 1082 (403 patients and 679 controls) unrelated subjects using TaqMan assays. The results showed that the genotypes, alleles, and haplotypes of the three SNPs were not significantly different between the schizophrenic patients and healthy controls either in the overall analysis or in the gender-stratified analysis (all *p* > 0.05). However, we did find a significant effect of the rs2868371 genotype on the age of onset, negative symptoms, and disorganized symptoms in the five-factor model of PANSS (all *p* < 0.01). Post hoc comparisons showed that carriers of the rs2868371 G/G genotype had significantly higher negative and disorganized factor scores than those with the C/G and C/C genotypes, respectively. Further investigations with other larger independent samples are required to confirm our findings and to better explore the effect of the *HSPB1* polymorphisms on the risk and symptomatology of SCZ.

## 1. Introduction

Schizophrenia (SCZ) is a common and severe psychiatric illness that affects 0.5–1% of the population in late adolescence or early adulthood. It has long been known that SCZ has a complex inheritance with a substantial genetic component [1]. The genetic architecture of SCZ has now been established as being highly polygenic with hundreds of common risk variants with small individual effects in combination with rare variants and copy number variants with larger effects that contribute to the disease risk [2–4]. Multiple candidate gene association studies on many ethnic groups have been published over the years and have supported the established hypotheses about the etiology of SCZ (dysfunction of neurotransmission, disruption of early brain development, and dysregulation of the immune system) [5]. However, most of these studies have reported inconsistent, unreplicable findings partly because of the differences in the underlying genetic architecture across various populations [6]. In recent years, genome-wide association studies (GWASs) have identified many new susceptible regions and/or genes for SCZ, and most of the well-studied candidate genes have failed to exhibit any associations in GWASs [7]. Most GWASs have been performed in populations of a European ancestry [8–10] with relatively few studies in other populations [11, 12]. The latest and the largest GWAS conducted to date identified associations with SCZ at 270 independent loci [10]. The GWASs findings were supported by pathway analyses of the SCZ risk loci that found that the associations were concentrated in the genes that are involved in neuron differentiation, synaptic plasticity, and neurotransmission [9, 13].

Heat shock proteins (HSPs) are a large family of highly conserved molecular chaperones that play pivotal roles in the maturation, refolding, and degradation of proteins. HSPs have been grouped into several main families based on a comparable molecular weight: HSP100, HSP90, HSP70, HSP60, HSP40, and small HSPs (sHSPs). Some HSPs are constitutively expressed in most cells, while others are highly inducible following a wide range of stressors, including ischemia, hyperthermia, and oxidative stress [14, 15].

Ten members of sHSPs (HSPB1 to HSPB10) have been identified in the human genome and have been categorized as Class I or Class II (reviewed in [16]). HSPB1 (HSP27) belongs to the class I sHSPs, which are widely distributed and predominantly stress-inducible, and which play an important role in cell survival under stress conditions. In addition to the HSP70 family members, HSP27 seems to be another attractive subject of interest in the field of psychiatric disorders such as SCZ. Previous studies have identified changes in the expression of *HSPB1* in SCZ patients [17, 18]. HSP27 is primarily known for its role in protecting neurons against damage by different stressors at vulnerable stages of development [19]. It has been suggested that an aberrant expression of inducible HSPs could be the cause of early brain abnormalities in the developmental processes that result in SCZ [20]. HSP27 is also expressed in temporally and spatially controlled patterns during the development of the nervous system. There is growing amount of evidence that HSP27 plays a key role in processes such as neuronal differentiation and neurite outgrowth [15, 21, 22]. Many of the genes that have been associated with SCZ exhibit a preferential expression in brain development [23]. The neuroprotective function of HSP27 has been well established in recent years. HSP27 has been found to associate with the protein aggregates in many neurodegenerative diseases, including Alzheimer's disease, amyotrophic lateral sclerosis, and Parkinson's disease [22, 24]. An overexpression of HSP27 is well documented to significantly augment neuronal survival in animal models of these diseases *via* its chaperoning activity or the direct inhibition of the apoptotic pathways [21]. A number of cytoprotective effects have also been shown using the HSP27 transgenic lines and in viral transfection experiments. The overexpression or adenoviral delivery of HSP27 confers protection against ischemia, kainate-induced neuronal apoptosis in the hippocampus or nerve injury-induced death [14, 21, 24]. Finally, mutations in the *HSPB1* gene are known to cause hereditary neuropathies [16].

Despite the wide range of essential functions that are played by HSPB1 in the CNS, almost all of the reported case-control association studies in SCZ have been focused on the HSP70-encoding genes, especially *HSPA1A*, *HSPA1B*, and *HSPA1L* [25–30].

In this report, we aimed to determine whether the *HSPB1* polymorphisms (rs2868370, rs2868371, and rs7459185) contribute to the risk of developing SCZ in a Polish population. Furthermore, we also examined the association between the *HSPB1* polymorphisms and clinical characteristics, including the age of onset, the symptoms as determined by the PANSS, and suicidal behavior. To the best of our knowledge, this is the first study that has investigated the association between the *HSPB1* polymorphisms and SCZ.

## 2. Materials and Methods

### 2.1. Subjects

A total of 1082 (403 SCZ patients and 679 controls) unrelated Caucasians of a Polish origin living in the same geographic area (Upper Silesia) were recruited for this study. There were 162 (40%) women and 241 (60%) men (mean age 41.5 ± 12.4) in the SCZ group and 320 (47%) women and 359 (53%) men (mean age 40.4 ± 8.8) in the control group.

The participants with SCZ were recruited from inpatients who were being treated at the Department and Clinic of Psychiatry, Medical University of Silesia in Katowice and the Neuropsychiatric Hospital in Lubliniec. All of the recruited patients met the following inclusion criteria: (I) Those who were diagnosed with paranoid schizophrenia based on the Diagnostic and Statistical Manual of Mental Disorders, Fourth Edition, Text Revision (DSM-IV-TR) criteria with the diagnosis confirmed by two experienced psychiatrists. As a diagnostic tool, the Structured Clinical Interview for DSM-IV Axis I Disorders, Clinical Version (SCID-I-CV) was used [31]. (II) Those who were hospitalized due to an acute exacerbation of schizophrenia. (III) Those who did not have any other Axis I and Axis II disorders, neurological illness, or endocrine or autoimmune diseases. The Positive and Negative Syndrome Scale (PANSS) [32] was used by experienced psychiatrists to assess the severity of the symptoms of the patients at the time of their admission. The factors were calculated using the classic PANSS, which is composed of 30 items that are divided into three subscales: positive (items P1 to P7), negative (items N1 to N7), and general psychopathology (items G1 to G16), as well as using the five-factor model of the PANSS by van der Gaag et al. [33], which includes the following factors: positive (total score of P1+P3+G9+P6+P5+G1+G12+G16-N5), negative (total score of N6+N1+N2+N4+G7+N3+G16+G8+G13-P2), disorganized (total score of N7+G11+G10+P2+N5+G5+G12+G13+G15+G9), excitement (total score of G14+P4+P7+G8+P5+N3+G4+G16), and emotional (total score of G2+G6+G3+G4+P6+G1+G15+G16), which included all 30 of the PANSS items. The age of onset was defined as the age at which the positive symptoms first appeared. The clinical details (data on the age of onset, family history of mental health, and suicidal behavior) were obtained from a patient's medical records, during the clinical exam or were provided by family members.

The control subjects were healthy volunteer blood donors who were recruited from the Regional Centre of Blood Donation and Treatment in Katowice. The control volunteers were matched to the patients for age and gender. Based on a questionnaire and an interview, healthy controls with a personal or family history of mental illness, suicide attempts, a history of substance abuse or dependency, any other neurological disorders, chronic and acute physical illness such as infection, or autoimmune or allergic diseases were excluded.

The objective and procedure of this project were explained to all of the included participants, and all of the participants signed informed consent forms. This study was conducted in accordance with the Declaration of Helsinki and approved by the Bioethics Committee of the Medical University of Silesia (no. KNW/0022/KB1/38/I/12).

### 2.2. SNP Selection Criteria

Three SNPs (rs2868370 G/A, rs2868371 C/G, and rs7459185 G/C) of the *HSPB1* gene were selected to be examined. The selected SNPs met all of the following criteria: (1) a minor allelic frequency (MAF) of at least 10% in the European population (SNP information was retrieved from the National Center for Biotechnology Information, dbSNP, http://www.ncbi.nlm.nih.gov/SNP/) (low MAF SNPs are more susceptible to genotyping errors and they also have low power to detect any association for a given effect size; (2) potentially functional variants that are located in the promoter, regulatory or untranslated regions of the *HSPB1*; and (3) availability for TaqMan SNP Genotyping Assay.

**Figure 1:**
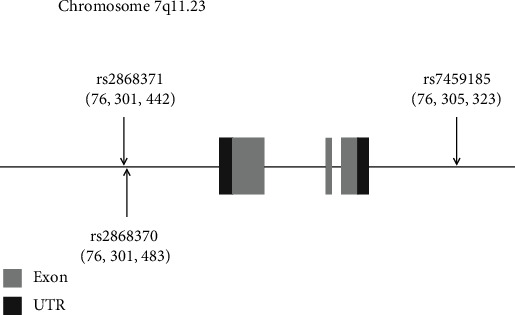
Schematic diagram of the positions of three examined *HSPB1* SNPs using NCBI database. *HSPB1* is located on chromosome 7q11.23. The arrows and the numbers in parentheses indicate the locus of each SNP. rs2868370 and rs2868371 locate in the promoter and rs7459185 locates on the near 3′UTR.

The locations of the *SNPs* that were genotyped are presented in Figure 1. rs2868370 and rs2868371 locate in the promoter of *HSPB1* and rs7459185 locates on the near 3′UTR of *HSPB1*. Additionally, evidence from the literature suggests a potential functional effect of rs2868371 SNP on gene expression [34].

### 2.3. Genotyping Analysis

Genomic DNA was extracted from whole peripheral blood samples that were collected with the anticoagulant EDTA using a QIAamp DNA Blood Mini Kit (Qiagen, Valencia, CA, USA) according to the standard protocol. The DNA concentration and purity were measured using a BioPhotometer plus (Eppendorf AG, Hamburg, Germany).

The rs2868370, rs2868371, and rs7459185 SNPs were genotyped using the allele-specific TaqMan assays on a CFX96 real-time PCR detection system (Bio-Rad), in a 96-well format. The real-time PCR reactions were performed in a final volume of 25 *μ*L in each well of an optical plate that contained 10 ng of the genomic DNA, 12.5 *μ*L of the TaqMan Universal PCR Master Mix (Applied Biosystems), 1.25 *μ*L of the combined primers and probes mix (Applied Biosystems), and nuclease free water. The cycle conditions were 95°C for 10 min, 95°C for 15 s, and 60°C for 1 min. The last two steps were repeated 40 times. Two nontemplate controls were also run for each analysis along with the three previously genotyped control samples, which represented specific genotypes for each SNP. Commercially available allele-specific TaqMan primers and probes were used (Applied Biosystems). The catalog numbers for the rs2868370, rs2868371, and rs7459185 polymorphisms were C_16146174_10, C_16146175_10, and C_2668492_10, respectively.

To ensure the quality, approximately 5% of the samples were repeated in order to validate the results of the genotyping and the concordance was 100%. The genotyping call rates in the entire sample were 99%. Samples with missing genotypes were removed from the analysis. Furthermore, the genotype frequencies were similar to the CEU sample frequencies (available on the NCBI website).

### 2.4. Statistical Analysis

STATISTICA 13.0 PL (StatSoft, TIBCO Inc., Palo Alto, CA, USA), Stata SE 13.0 (StataCorp LP, Collage Station, TX, USA), and R software [35] were used to perform the statistical analyses. All of the tests were two-tailed, and the level of statistical significance was adjusted to *p* < 0.05. Imputations were not performed for any missing data. The nominal and ordinal data are expressed as percentages, while the descriptive variables are expressed as the mean value ± standard deviation. The distribution of the variables was evaluated using the Shapiro-Wilk test and a quantile-quantile (Q-Q) plot, and the homogeneity of the variances was assessed using the Levene test. The Hardy-Weinberg equilibrium (HWE) was examined using Fischer's exact test. The allele frequencies and genotype distribution were assessed using the Chi-square (*χ*^2^) test and the maximum likelihood *χ*^2^ test. The linkage disequilibrium (LD) patterns and haplotypes were evaluated using SNPStats. Five inheritance models (codominant, dominant, recessive, overdominant, and log-additive) were also used to assess any potential association with the SCZ risk and the best fitting models were determined using the Akaike information criterion (AIC) and Bayesian information criterion (BIC). Finally, a two-way ANOVA (sex and genotype) with Tukey's post-hoc test was used to examine the effect of the genotypes on the clinical variables.

## 3. Results

The initial analysis showed a significant difference between the males and females in the study group with respect to age (38.6 ± 11.8 vs. 45.8 ± 12.2, *p* < 0.001). There was no difference in age between the males and females among the controls (40.7 ± 9.3 vs. 40.2 ± 8.3, *p* = 0.53).

### 3.1. The HWE Analysis

The distributions of the genotypes of the *HSPB1* variants were in the Hardy-Weinberg equilibrium for all of the groups (patients: rs2868370 (*p* = 0.30), rs2868371 (*p* = 0.26), and rs7459185 (*p* = 0.07); controls: rs2868370 (*p* = 0.71), rs2868371 (*p* = 0.85), and rs7459185 (*p* = 0.71)); males (patients: rs2868370 (*p* = 0.19), rs2868371 (*p* = 0.51), and rs7459185 (*p* = 0.09); controls: rs2868370 (*p* = 0.62), rs2868371 (*p* = 0.35), and rs7459185 (*p* = 0.37)); and females (patients: rs2868370 (*p* = 0.92), rs2868371 (*p* = 0.43), and rs7459185 (*p* = 0.42); controls: rs2868370 (*p* = 0.98), rs2868371 (*p* = 0.18), and rs7459185 (*p* = 0.78)).

### 3.2. The Genotype and Allele Analyses

Table 1 lists the genotype distributions and allele frequencies of the three studied *HSPB1* SNPs in the SCZ patients and healthy controls. There were no significant differences in the genotype distributions and allele frequencies between the patients and controls according to the *χ*^2^ test. Additionally, there were no significant differences in either genotype or allele distributions between the patients and controls in the gender-stratified analysis. We also performed an analysis in the subgroups of patients with a positive or a negative family history of SCZ. There was a positive family history for 98 individuals who had affected first-degree or second-degree relatives in our study population. No significant differences were observed between these subgroups.

We further examined the different genetic models of inheritance (codominant, dominant, recessive, overdominant, and log-additive) in order to assess the potential association between the *HSPB1* polymorphic sites and the SCZ risk. Unfortunately, the inheritance modeling did not indicate any significant differences in the genotype or allele distributions of any of the three SNPs between the controls and patients (all *p* > 0.05). The results were also not significant in the gender-stratified models (data not shown).

### 3.3. The LD and Haplotype Analyses

The linkage disequilibrium (LD) analysis showed a strong LD between rs2868370 and rs2868371 (*D*′ = 0.999, *r*^2^ = 0.088, *p* < 0.001), moderate-strong LD between rs2868370 and rs7459185 (*D*′ = 0.7323, *r*^2^ = 0.3236, *p* < 0.001), and no LD between rs2868371 and rs7459185 (*D*′ = 0.0271, *r*^2^ = 0.0001, *p* = 0.63). A slightly different pattern was observed in the female sample: rs2868371 and rs7459185 were in a weak LD (*D*′ = 0.1882, *r*^2^ = 0.0053, *p* < 0.05).

The results of the haplotype analysis are listed in Table 2. Six haplotypes of the three SNPs were estimated to have a frequency of >1%. There were no significant differences in the haplotype frequencies between the patients and the controls in either the entire sample or in the male and female subgroups.

### 3.4. Analysis of the Impact of *HSPB1* Variants on the Clinical Parameters

A two-way ANOVA (sex×genotype) was performed to assess the relationship between the individual *HSPB1* SNPs (rs2868370, rs2868371, and rs7459185) and the age of onset and the severity of the symptoms as measured by the PANSS (Table 3). There were no significant differences in the total or three subscale scores for the PANSS (positive, negative, and general) when the different genotypes were compared (data not shown). When a five-factor model of the PANSS by van der Gaag et al. [33] was used, the difference was significant among the genotypes of rs2868371 in the negative factor scores (*F* = 5.44, *η*^2^ = 0.0267, *p* < 0.01) and the disorganized factor scores (*F* = 5.58, *η*^2^ = 0.0273, *p* < 0.01). For the negative factor, Tukey's post hoc test showed significant differences between the C/C and C/G female carriers (*p* < 0.05), C/C and G/G female carriers (*p* < 0.05), and C/C and G/G male carriers (*p* < 0.05) and a trend toward significance when the males with the C/C and C/G genotypes were compared (*p* = 0.067). We observed that the females and males who carried the rs2868371 G/G genotype had higher mean PANSS negative scores than the females and males who carried the rs2868371 C/C genotype (females: 27.7 vs. 23.7; males: 26.1 vs. 23.2). For the disorganized factor, Tukey's post hoc test showed significant differences between the C/C and G/G female carriers (*p* < 0.05) and the C/C and G/G male carriers (*p* < 0.05). We observed that the females and males who carried the rs2868371 G/G genotype had higher mean PANSS disorganized scores than the females and males who carried the rs2868371 C/C genotype (females: 35.5 vs. 32.6; males: 35.3 vs. 31.9). Additionally, there was a significant main effect of the rs2868371 genotype on the age of onset (*F* = 4.92, *η*^2^ = 0.0242, *p* < 0.01). Tukey's post hoc test showed significant differences between the C/C and C/G female carriers (*p* < 0.05) and the C/C and C/G male carriers (*p* < 0.05).

We further investigated the association between the *HSPB1* polymorphic sites and suicidal behavior in the all of the sample and gender-stratified subgroups. We did not find any significant associations (all *p* > 0.05). We also did not detect any significant associations between a positive or negative family history of SCZ and suicidal risk.

## 4. Discussion

In the presented case-control study, we attempted to investigate whether three SNPs (rs2868370, rs2868371, and rs7459185) within *HSPB1* are associated with the risk and clinical features of SCZ in a Polish Caucasian population. To the best of our knowledge, this is the first study that investigated the associations between the *HSPB1* polymorphisms and SCZ. We found that rs2868371 SNP has an impact on the age of onset and negative/disorganized symptoms of the disease. However, the results also revealed that there were no significant differences in the frequency of the genotypes, alleles, or haplotypes between the SCZ patients and the healthy controls in both the overall analysis and in the analyses that were stratified by gender or by a positive/negative family history of the disease.

Genetic association studies of SCZ are often hampered by the heterogeneity of the samples, which can limit the ability to detect the true risk variants [36]. Therefore, it is important to reduce this heterogeneity by creating more genetically homogenous subgroups of patients. Our study population was highly homogenous ethnically (Polish Caucasians from Upper Silesia), with respect to the SCZ subtype (patients with a diagnosis of paranoid schizophrenia exclusively), and none of the patients were newly diagnosed in order to prevent any discrepancies in the diagnosis and clinical measures. Additionally, we used a consensus five-factor model for interpreting the PANSS that is thought to be more representative of the clinical symptoms of SCZ patients than the original three subscale PANSS. Moreover, we also performed all of the analyses in the subgroups stratified by gender. Gender differences in SCZ are one of the most consistently reported aspects of the disease. These include differences in the age of onset, premorbid functioning, clinical presentation, course and treatment response [37]. A number of association studies have also demonstrated the gender effect on SCZ at the genetic level [38–41].

The two selected polymorphisms (rs2868370 and rs2868371) are located in the promoter of *HSPB1* gene. There is evidence that suggests that the SNPs that are located within the promoter regions have a high potential to alter gene expression by affecting the binding of the transcription factors [42, 43]. The functionality of the rs2868371 was confirmed in a previous study. A luciferase reporter assay demonstrated that the rs2868371 C allele decreased the expression of HSP27 in the studied cell lines [34]. The third selected SNP (rs7459185) is a downstream gene variant that is located on the near 3′UTR. Genetic variations in the 3′UTRs of genes can alter their expression by affecting miRNA binding and mRNA stability [42, 44, 45]. Although rs7459185 is outside of the 3′UTR sequence, a bioinformatics analysis showed a decrease in the *HSPB1* mRNA expression levels in the presence of the rs7459185 CC genotype [46].

At present, our study found no association between the genotype, allele, or haplotype distribution of the *HSPB1* polymorphisms and the risk of SCZ. To the best of our knowledge, this is the first study that investigated the association between the *HSPB1* variants and SCZ, and therefore, we cannot directly relate our findings to the results from previous studies. To date, only a few polymorphisms of the HSP70 genes have been found to be significantly associated with the SCZ risk in studies that have mainly focused on three genes: *HSPA1A*, *HSPA1B*, and *HSPA1L*. Initial analyses with positive findings were performed on a Korean population [25, 26]. More recently, the case-control studies performed by our group in a Polish population provided evidence that the *HSPA1A* (rs1043618) and *HSPA1B* (rs539689) polymorphisms might be involved in the susceptibility to SCZ in a sex-dependent manner [28, 30].

SCZ is a highly polygenic disease with a remarkable phenotypic heterogeneity. Although *HSPB1* variation does not alter the susceptibility to SCZ, it might affect some of the features of SCZ. Therefore, we further analyzed the individual effects of the *HSPB1* polymorphisms (rs2868370, rs2868371, and rs7459185) on the age of onset and the severity of the symptoms, which were measured using the classic three-factor model of PANSS and the five-factor model of PANSS by van der Gaag et al. [33]. We found a significant effect of the rs2868371 genotype on the age of onset, the PANSS negative factor scores, and the PANSS disorganized factor scores (all *p* < 0.01). Post hoc comparisons showed that carriers of the rs2868371 G/G genotype (both males and females) had significantly higher negative and disorganized symptoms scores than those with C/G and C/C genotypes, respectively. Therefore, we speculated that the G/G genotype of the *HSPB1* rs2868371 polymorphism might act as a causative factor for the severity of the negative and disorganized symptoms in SCZ. Interestingly, the associations between the HSP70 gene polymorphisms and the different clinical variables of SCZ were common findings in our previous studies. Two polymorphisms, *HSPA1A* rs1043618 and *HSPA1B* rs1061581, were found to be significantly associated with positive PANSS scores. In the same study, significant differences were also observed in the age of onset between males and females among the *HSPA1A* rs1043618 genotypes [28]. We also detected the influence of the *HSPA1B* rs539689 genotype on the positive, general, and total PANSS scores and the association between the *HSPA1B* rs9281590 genotype, gender, and the general PANSS scores [30]. Finally, *HSPA1A* rs562047 was significantly associated with the PANSS total and PANSS negative scores [29]. Pae et al. [27] also found significant associations between the HSP70 gene polymorphisms and the clinical presentation and drug response in Korean SCZ patients. The mechanisms beyond the associations between the HSP gene polymorphisms and the clinical features of SCZ remain unclear, and some biological explanations for these associations are required in the future. Pae et al. [27] speculated that a HSP variation may differentially contribute to the negative and positive symptoms of SCZ in accordance with the molecular functions of these proteins.

HSPB1 is induced during cellular stress and contributes to neuron survival through its chaperoning, antioxidant, and antiapoptotic properties [14]. It might also contribute to the repair and protective mechanisms at a synapse following exposure to stress [47], which permits the normal neurotransmission in the brain to be preserved. Disturbances in multiple neurotransmitter systems have been linked to the development of the characteristic symptoms of SCZ. In this regard, a decreased HSPB1 expression may impair its protective functions under stress conditions. We found that the rs2868371 G/G genotype was associated with higher negative and disorganized symptoms scores compared to the C/G and C/C genotypes. The oxidative stress was reported to be higher in the SCZ patients, and the disruption in the oxidative balance was associated with the increased severity of negative symptoms [48]. Additionally, patients with the subtypes of SCZ who had predominantly negative symptoms exhibited a greater decrease in their glutathione levels compared to those with the other subtypes [49]. HSPB1 protects against oxidative stress by decreasing the reactive oxygen species by upholding the levels of glutathione in its reducing form [50]. Therefore, we hypothesized that the downregulation of *HSPB1* that is triggered by the rs2868371 G risk allele might attenuate cellular protection against oxidative stress, which may partly explain the biological mechanisms that underlie the observed associations. A previous study found that rs2868371 SNP affects the expression of *HSPB1* in normal bronchial epithelial cells with the G allele shown to exhibit a higher transcriptional activity than the C allele in a reporter assay [34]. Such an increased promoter activity of the rs2868371 G allele is inconsistent with phenotypic results in our study. On the other hand, gene expression levels are known to vary across different tissues despite the presence of the same genetic variation in these tissues. Fu et al. [51] observed that the SNPs that are located in the transcriptional regulatory elements and that associated with complex traits are more likely to affect gene expression in a tissue-dependent manner. Among the different mechanisms of tissue-dependent gene expression regulation, the opposite allelic direction mechanism (alleles have opposing effects on gene expression between tissues) was indicated. The authors stressed the great importance of investigating disease-relevant tissues in order to correctly characterize the functional effects of the disease-associated gene variants.

*HSPB1* together with 69 other genes has been indicated as potentially being associated with suicidal behavior in SCZ based on differences in its expression between the suicide and nonsuicide SCZ groups [52]. The current study failed to demonstrate any associations between the *HSPB1* variants and suicidal behavior in either the entire sample or in the gender-stratified analysis. However, we did find a strong significant association between the *HSPA1B* rs539689 polymorphism and attempted suicide in patients with SCZ in our previous study [30]. The C/C genotype and C allele were protective against suicidal behavior in all of the samples as well as in the male and female subgroups.

This study has several limitations that should be noted. First, our sample size was modest, especially for the purposes of the stratified analyses. Therefore, our findings should be confirmed through studies using a larger sample size. Second, the three polymorphisms that were selected for the analysis might not be substantially informative due to their incomplete coverage of the *HSPB1* and further studies should be done to analyze more variants. Third, although stratified analyses were performed, more subtle stratified analyses should be used in further studies, e.g., in subgroups of patients with early-onset and late-onset SCZ. Fourth, despite the influence of the genotypes of the rs2868371 SNP on the SCZ psychopathology and age of onset, the specific mechanism remains unclear.

## 5. Conclusions

We analyzed three *HSPB1* variants (rs2868370, rs2868371, and rs7459185) in patients with SCZ in a Polish Caucasian population. Our study revealed that the *HSPB1* rs2868371 polymorphism might affect clinical features of SCZ as this SNP was significantly associated with the PANSS negative and disorganized symptoms and the age of onset. However, further investigations in other independent larger samples in different ethnicities are required to confirm our findings and to better explore the effect of the *HSPB1* polymorphisms on the risk and symptomatology of schizophrenia.

## Figures and Tables

**Table 1:** Comparison of the allele frequencies and genotype distributions of the three *HSPB1* SNPs between the SCZ cases and controls in all, women, and men samples.

dbSNP	Genotype/allele	All		*χ* ^2^	*p*	Women		*χ* ^2^	*p*	Men		*χ* ^2^	*p*
		Cases	Controls			Cases	Controls			Cases	Controls		
rs2868370	G/G	276 (68.5%)	442 (65%)	2.01	0.37	111 (69%)	212 (66%)	0.26	0.88	165 (68%)	230 (64%)	2.49	0.29
	G/A	111 (27.5%)	214 (31.5%)			46 (28%)	97 (30%)			65 (27%)	117 (33%)		
	A/A	16 (4%)	23 (3.5%)			5 (3%)	11 (3%)			11 (5%)	12 (3%)		
	G	663 (82%)	1098 (81%)	0.66	0.42	268 (83%)	521 (81%)	0.25	0.62	395 (82%)	577 (80%)	0.47	0.49
	A	143 (18%)	260 (19%)			56 (17%)	119 (19%)			87 (18%)	141 (20%)		
rs2868371	C/C	208 (52%)	347 (51%)	0.99	0.61	82 (51%)	155 (48%)	0.24	0.89	126 (52%)	192 (53%)	1.61	0.45
	C/G	170 (42%)	279 (41%)			70 (43%)	143 (45%)			100 (41%)	136 (38%)		
	G/G	25 (6%)	53 (8%)			10 (6%)	22 (7%)			15 (6%)	31 (9%)		
	C	586 (73%)	973 (72%)	0.28	0.6	234 (72%)	453 (71%)	0.22	0.64	352 (73%)	520 (72%)	0.05	0.82
	G	220 (27%)	385 (28%)			90 (28%)	187 (29%)			130 (27%)	198 (28%)		
rs7459185	G/G	226 (56%)	352 (52%)	2.36	0.31	90 (56%)	166 (52%)	0.93	0.63	136 (56%)	186 (52%)	1.38	0.50
	G/C	142 (35%)	271 (40%)			59 (36%)	131 (41%)			83 (34%)	140 (39%)		
	C/C	35 (9%)	56 (8%)			13 (8%)	23 (7%)			22 (9%)	33 (9%)		
	G	594 (74%)	975 (72%)	0.92	0.34	239 (74%)	463 (72%)	0.22	0.64	355 (74%)	512 (71%)	0.79	0.37
	C	212 (26%)	383 (28%)			85 (26%)	177 (28%)			127 (26%)	206 (29%)		

**Table 2:** The results of the haplotype analysis with the three SNPs (rs2868371–rs2868370–rs7459185) of the *HSPB1* in the SCZ patients and controls.

Haplotype	All			Women			Men		
	Frequency	OR (95% CI)	*p*	Frequency	OR (95% CI)	*p*	Frequency	OR (95% CI)	*p*
C–G–G	0.4877	1.00	—	0.4779	1.00	—	0.4947	1.00	—
G–G–G	0.2014	0.92 (0.72-1.18)	0.52	0.2174	0.92 (0.63-1.35)	0.67	0.1894	0.94 (0.68-1.31)	0.71
C–A–C	0.1503	0.87 (0.67-1.12)	0.28	0.1487	0.85 (0.56-1.29)	0.44	0.1516	0.87 (0.62-1.22)	0.42
G–G–C	0.0782	0.89 (0.62-1.27)	0.52	0.0699	0.85 (0.45-1.58)	0.60	0.0839	0.90 (0.58-1.40)	0.63
C–G–C	0.0465	0.96 (0.59-1.57)	0.88	0.0532	1.15 (0.57-2.34)	0.69	0.042	0.85 (0.43-1.66)	0.63
C–A–G	0.0360	0.95 (0.57-1.60)	0.86	0.0329	1.06 (0.47-2.39)	0.90	0.0384	0.88 (0.45-1.72)	0.71

OR: odds ratio; CI: confidence interval.

**Table 3:** Results from the two-way ANOVA (sex and genotype) on clinical variables in the schizophrenic group.

Variable	rs2868370 G/A			rs2868371 C/G			rs7459185 G/C		
	Sex	Genotype	Sex×genotype	Sex	Genotype	Sex×genotype	Sex	Genotype	Sex×genotype
Age of onset	0.953	0.813	0.568	0.870	0.008^a^^∗^	0.952	0.149	0.565	0.033
Five-factor PANSS^∗∗^									
Positive factor	0.659	0.615	0.394	0.515	0.429	0.947	0.027	0.758	0.095
Negative factor	0.317	0.441	0.444	0.367	0.005^b^^∗^	0.888	0.146	0.269	0.467
Disorganized factor	0.402	0.703	0.317	0.894	0.004^c^^∗^	0.323	0.528	0.338	0.412
Excitement factor	0.928	0.569	0.846	0.914	0.101	0.998	0.899	0.921	0.967
Emotional factor	0.751	0.679	0.613	0.418	0.362	0.606	0.955	0.163	0.977

Nominally significant *p* values are bolded. ^∗^The power of the test > 0.8. ^∗∗^van der Gaag's model. ^a^Mean age of onset (years): women—C/C 26.8 ± 6.8, C/G 24.3 ± 6.6, and G/G 24.9 ± 6.3; men—C/C 26.6 ± 7.3, C/G 24.5 ± 6.5, and G/G 25.5 ± 8.9. ^b^Mean negative PANSS factor scores: women—C/C 23.7 ± 5.7, C/G 22.9 ± 5.6, and G/G 27.7 ± 5.4; men—C/C 23.2 ± 5.9, C/G 22.6 ± 5.6, and G/G 26.1 ± 8.0. ^c^Mean disorganized PANSS factor scores: women—C/C 32.6 ± 6.9, C/G 30.1 ± 6.9, and G/G 35.5 ± 7.6; men—C/C 31.9 ± 6.7, C/G 31.5 ± 6.6, and G/G 35.3 ± 8.5.

## Data Availability

All data generated or analyzed during the current study are within this paper or are available from the corresponding author on a reasonable request.
